# Data on treatment of nafcillin and ampicillin antibiotics in water by sonochemistry

**DOI:** 10.1016/j.dib.2020.105361

**Published:** 2020-03-02

**Authors:** Diana M. Montoya-Rodríguez, Yenny Ávila-Torres, Efraím A. Serna-Galvis, Ricardo A. Torres-Palma

**Affiliations:** a Grupo de Investigación en Remediación Ambiental y Biocatálisis (GIRAB), Instituto de Química, Facultad de Ciencias Exactas y Naturales, Universidad de Antioquia UdeA, Calle 70 No. 52-21, Medellín, Colombia; b Grupo de Investigación QUIBIO, Facultad de Ciencias Básicas, Universidad Santiago de Cali, Santiago de Cali, Pampalinda, Colombia

**Keywords:** β-Lactam antibiotics, Combination of processes, Matrix effect, Sonochemistry, Water treatment

## Abstract

Ampicillin and nafcillin antibiotics were treated by high frequency ultrasound (at 375 kHz and 24.4 W). Degradations followed pseudo-first order kinetics, which constants were k: 0.0323 min^−1^ for AMP and k: 0.0550 min^−1^ for NAF. Accumulation of sonogenerated hydrogen peroxide and inhibition degree of sonochemical removal (IDS) in presence of a radical scavenger were also stablished. Afterwards, ultrasound was combined with UVC light (sono-photolysis), with ferrous ion (sono-Fenton), and with ferrous ion plus UVC light (sono-photo-Fenton) to degrade the antibiotics. Furthermore, treatment of the pollutants in a complex matrix and removal of antimicrobial activity (AA) were considered. The antibiotics evolution was followed by HPLC-DAD technique and the accumulation of sonogenerated H_2_O_2_ was measured by an iodometry-spectrophotometry methodology (77.6 and 57.3 μmol L^−1^ of H_2_O_2_ after 30 min of sonication were accumulated in presence of AMP and NAF, respectively). IDS was analyzed through treatment of the antibiotics in presence of 2-propanol (87.1% for AMP and 56 % for NAF) and considering the hydrophobic character of pollutants (i.e., Log P values). Antimicrobial activity evolution was assessed by the Kirby-Bauer method using *Staphylococcus aureus* as indicator microorganism (sono-photo-Fenton process removed 100% of AA after 60 and 20 min for AMP and NAF, respectively). Finally, for degradations in the complex matrix, a simulated effluent of municipal wastewater treatment plant was utilized (sono-photo-Fenton led to degradations higher than 90 % at 60 min of treatment for both antibiotics). The data from the present work can be valuable for people researching on treatment of wastewaters containing antibiotics, application of advanced oxidation technologies and combination of sonochemical process with photochemical systems.

**Table:** Specifications Table

Subject	Environmental chemistry
Specific subject area	Advanced oxidation processes
Type of data	Table Figure
How data were acquired	Data were acquired by using high performance liquid chromatography (HPLC-DAD) and spectrophotometry.
Data format	Raw Analyzed
Parameters for data collection	Experiments were developed at fixed conditions. A Meinhardt ultrasound reactor was used. For the combined processes, the ultrasound reactor was complemented by an UVC-lamp with main emission at 254 nm of 4 W placed on a quartz sleeve (which was submerged in the aqueous sample). In all cases, reactor temperature was controlled using a Huber Minichiller and 250 mL of antibiotic solutions were treated.
Description of data collection	A comparative dataset for the sonochemical degradation of ampicillin (AMP) and nafcillin (NAF) is reported. Initially, the degradation in distilled water was done. To determine proximity of antibiotics to the cavitation bubble, the inhibition of degradation in presence of 2-propanol was analyzed. Then, the combination of ultrasound with Fenton-based processes was evaluated. Afterwards, the evolution of antimicrobial activity was tested for each process. Finally, the effect of matrix on the degradation of pollutants by sono-photo-Fenton process was evaluated. All experimental data were obtained at lab-scale.
Data source location	Universidad de Antioquia UdeA, Medellín, Colombia
Data accessibility	Mendeley data repository through the following link: https://data.mendeley.com/datasets/9g63wd42sn/draft?a&equals;a0e3350f-7244-49f7-be23-030fa701eecf

**Table:** 

**Value of the Data** • The data presents differences between degradation of NAF and AMP by high frequency ultrasound. The readers can recognize the structural effects, pollutants closeness to the cavitation bubble and hydrophobic character of the target antibiotics. • Data can benefit people working on treatment of wastewaters containing antibiotics. • Data can be useful for comparative purposes about elimination of antibiotics by coupling sonochemistry with iron (II) and UVC light (i.e., sono-photo-Fenton system). • Data could be useful for scaling up of the sonochemical process to treat organic pollutants in complex aqueous matrixes rich in hydrophilic components. • Data may be utilized in further experimental works and reviews on degradation of β-lactam antibiotics by advanced oxidation processes.

## 1 Data description

**Fig. 1:**
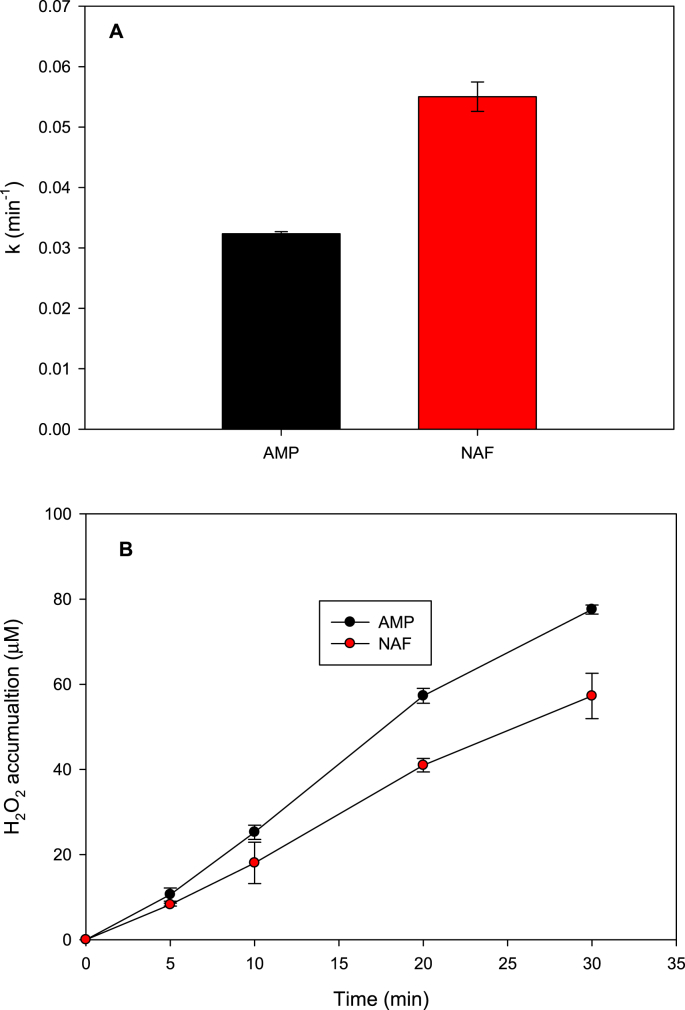
Individual treatment of ampicillin (AMP) and nafcillin (NAF) by sonochemistry. **A.** Pseudo-first order degradation constants (k). **B.** Hydrogen peroxide accumulation during degradation of the antibiotics by sonochemistry. Experimental conditions: [AMP]: [NAF]: 30 μM, power: 24.4 W, frequency: 375 kHz, initial pH: 6.5, volume: 250 mL, temperature: 20 ± 1 °C.

To determine the chemical structure effect of antibiotics, the individual elimination by sonochemistry (at 375 kHz of frequency and 24.4 W of actual power) of ampicillin (AMP) and nafcillin (NAF) was initially carried out in distilled water. The pollutants degradation followed a pseudo first-order kinetics; thus, the corresponding degradation constants (k) were calculated, which are shown in Fig. 1A [1].

It is well-known that the sonochemical process produces hydroxyl radical (HO·) through water cleavage (Eq. (1)). Hydroxyl radicals can attack organic pollutants (Eq. (2)) such as antibiotics, or combine themselves to form hydrogen peroxide (H_2_O_2_, Eq. (3)) [1]. Indeed, the accumulation of H_2_O_2_ during process is an indicator of sonochemical activity [2]. Fig. 1B shows the H_2_O_2_ evolution during the sonochemical treatment of NAF or AMP.

(1) H_2_O +))) → H^·^ + HO^·^

(2) HO^·^ + organic pollutants → degradation products

(3) 2 HO^·^ → H_2_O_2_

To test the closeness of the antibiotics to cavitation bubbles, the compounds were treated in presence of 2-propanol (100 times more concentrated than the pollutants) [3]. Then, inhibition degree of sonochemical degradation (IDS) was calculated according to Eq. (4) (based on the pseudo-first order constants for treatment in absence and presence of the scavenger). Table 1 presents the IDS values, which was 87 and 56% for AMP and NAF, respectively. Additionally, Table 1 contains the Log P values for both antibiotics (this parameter is related to the hydrophobic nature of organic pollutants, [4]).

(4) IDS = (k_in distilled water_-k_in 2-propanol presence_) ∗ 100 / k_in distilled water_

**Table 1:** Inhibition degree of sonochemical degradation (IDS) and octanol/water partition coefficient (Log P) for the antibiotics.

Antibiotic	IDS (%)	Log P^a^
Ampicillin	87.1	1.35
Nafcillin	56.0	3.30

a Log P values were taken from PubChem [5].

**Fig. 2:**
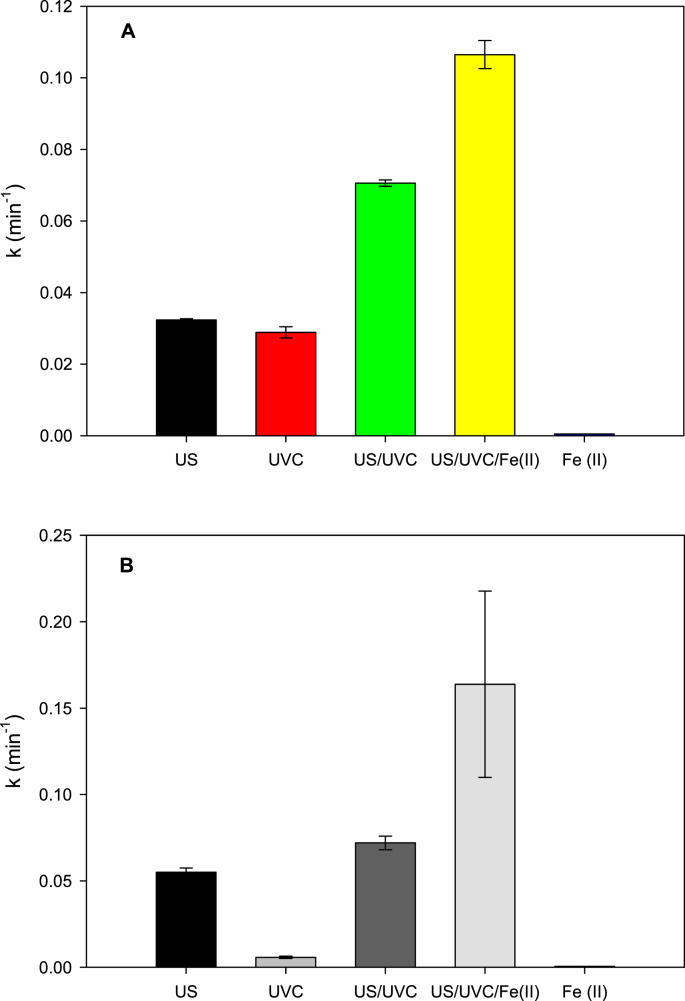
Degradation rate constants (k) for the processes combination. **A.** Case of AMP. **B.** Case of NAF. US: sonochemistry, UVC: photolysis by UV 254 nm, US/UVC: sono-photolysis, US/UVC/Fe(II): sono-photo-Fenton and Fe (II): action of iron (II) alone. Experimental conditions: [AMP]: [NAF]: 30 μM, [Fe^2+^]: 90 μM, UVC lamp: 4 W, actual ultrasound power: 24.4 W, frequency: 375 kHz, initial pH: 6.5, volume: 250 mL, temperature: 20 ± 1 °C.

A strategy to increase the degradation kinetics is the combination of ultrasound with other advanced oxidation processes [6]. Thus, in this work, ultrasound was combined with UVC light radiation (US/UVC, sono-photolysis) to promote extra formation of radicals through a homolysis sonogenerated hydrogen peroxide (Eq. (5)). Also, it was evaluated the addition of UVC plus ferrous ions to the sonochemical system (US/UVC/Fe(II), sono-photo-Fenton), with the purpose of increasing the amount of radical species by interaction of sonogenerated H_2_O_2_ with iron (generating a photo-Fenton process, (6), (7), (8) [7]. Moreover, control experiments (i.e., the individual degrading action of Fe (II) or UVC light) were also taken into account to determine the contribution of degradations due to iron (10), (11) and the photolysis of antibiotics (Eq. (11)). The data are given in terms of the pseudo-first order degradation constants for each process in Fig. 2.

(5) H_2_O_2_ + UVC → 2HO^·^

(6) Fe^2+^ + H_2_O_2_ → Fe^3+^ + HO^·^ + OH^−^

(7) Fe^3+^ + H_2_O + *hv* → Fe^2+^ + HO^·^ + H^+^

(8) Fe^3+^ + H_2_O_2_ + *hv* → Fe^2+^ + HO_2_^·^ + H^+^

(9) Fe^2+^ + O_2_ → Fe^3+^ + O_2_^·^^_^

(10) O_2_^·^^_^ + organic pollutants → degradation products

(11) UVC + organic pollutants → photodegradation products

**Fig. 3:**
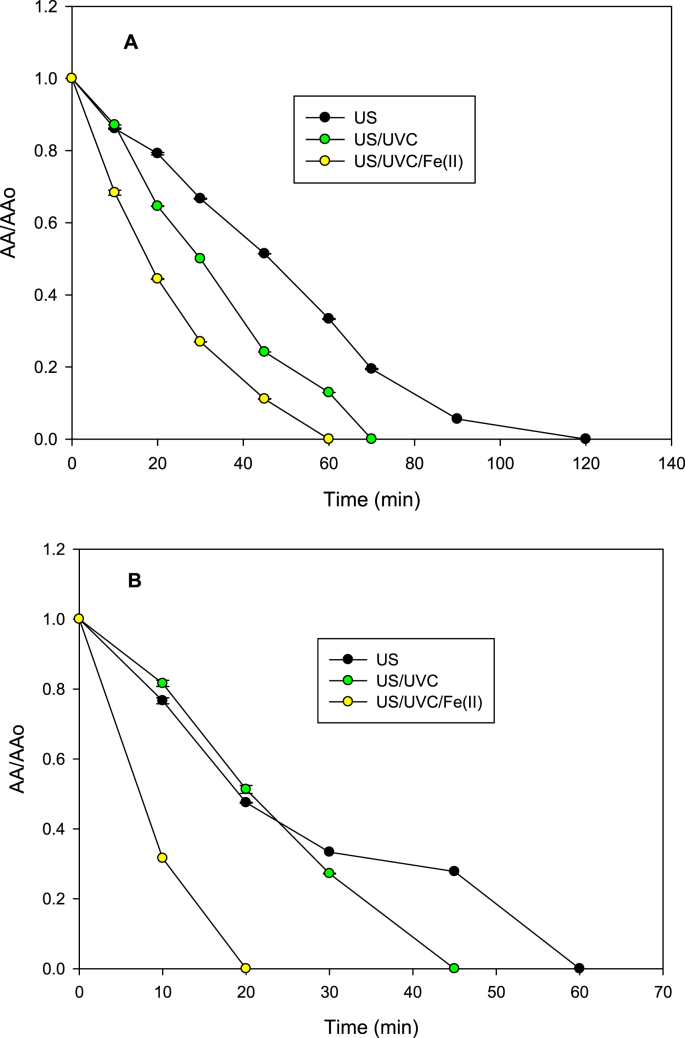
Elimination of antimicrobial activity (AA) against *S. aureus* by the different systems. **A.** Data for AMP. **B.** Data for NAF. US: sonochemistry, US/UVC: sono-photolysis and US/UVC/Fe(II): sono-photo-Fenton. Experimental conditions: [AMP]: [NAF]: 30 μM, [Fe^2+^]: 90 μM, UVC lamp: 4 W, actual ultrasound power: 24.4 W, frequency: 375 kHz, initial pH: 6.5, volume: 250 mL, temperature: 20 ± 1 °C.

One of the most important parameters to considerer during degradation of antibiotics is the evolution of antimicrobial activity (AA), due to in some cases, despite of antibiotic removal the activity can persist [8,9]. Thus, for AMP and NAF treatment by ultrasound (US), sono-photolysis (US/UVC) and sono-photo-Fenton (US/UVC/Fe(II)) processes, the evolution of AA was determined. Fig. 3 presents the data of the antimicrobial activity for each system.

**Fig. 4:**
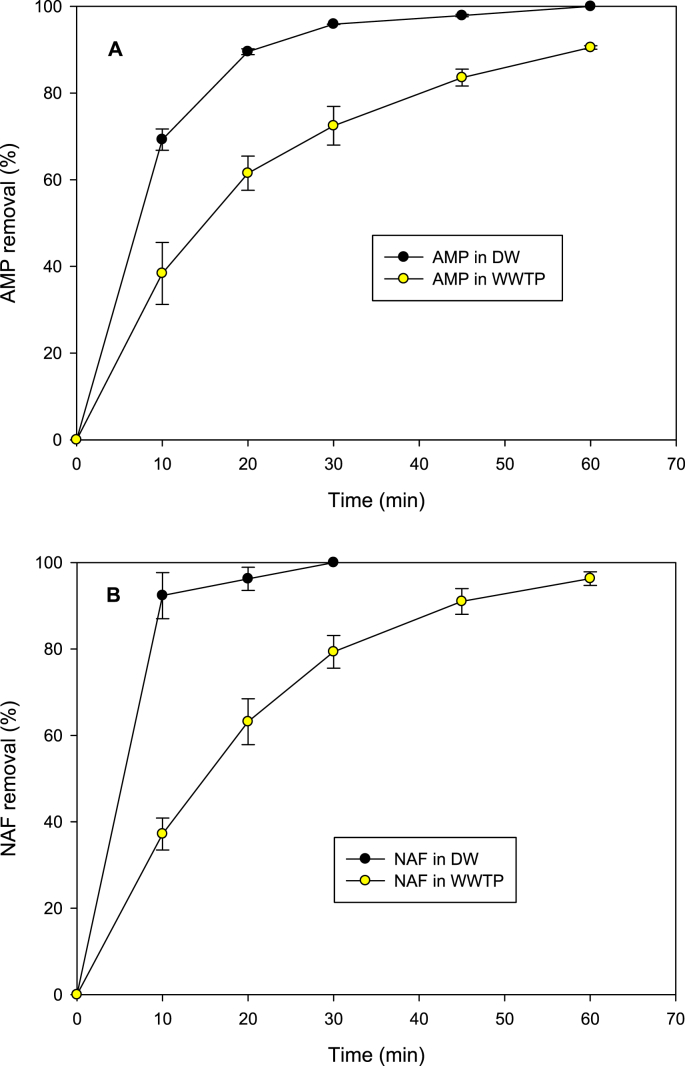
Comparison of antibiotics degradation in distilled water (DW) and in synthetic municipal wastewater treatment plant effluent (WWTP) by sono-photo-Fenton treatment. **A.** Case of AMP. **B.** Case of NAF. Experimental conditions: [AMP]: [NAF]: 30 μM, [Fe^2+^]: 90 μM, UVC lamp: 4 W, actual ultrasound power: 24.4 W, frequency: 375 kHz, initial pH: 6.5, volume: 250 mL, temperature: 20 ± 1 °C.

The individual elimination of the antibiotics in a complex matrix by sono-photo-Fenton system (which showed the best performance in Fig. 2, Fig. 3) was applied to a simulated effluent of wastewater treatment plant (WWTP, composition in Table 2). Fig. 4 compares the antibiotics removal in distilled water (DW) and in the complex matrix (WWTP) by the sono-photo-Fenton process.

**Table 2:** Composition of simulated effluent of wastewater treatment plant (WWTP, [10]).

Compound	Concentration (mg/L)	Concentration (μM)
NaCl	7	119
KCl	4	54
CaCl_2 ∗_ 2H_2_O	4	27
NaHCO_3_	96	1.142
CaSO_4_∗2H_2_O	60	348
MgSO_4_∗7H_2_O	125	507
K_2_HPO_4_	28	161
Urea	6	99.9
Peptone	32	–
Meat extract	22	–

-Not applicable.

Finally, it is presented the Table 3, which contains data from our research and previous works about degradation of AMP and NAF by others advanced oxidation processes (AOP).

**Table 3:** Data on AMP and NAF degradation by diverse AOP.

Antibiotic [reference]	AOP	Experimental conditions	Pseudo-first order constant (k)	Other relevant data
AMP [11]	Electrochemical oxidation	[AMP]: 50 mg L^−1^ BDD anode/GDE cathode Current density: 5 mA cm^−2^ [Na_2_SO_4_]: 0.05 M pH: 2.8 Volume: 250 mL	0.549 min^−1^ (9.15 x 10^−3^ s^−1^)	• 32% of mineralization after 120 min of treatment.
AMP [11]	Electro-Fenton	[AMP]: 50 mg L^−1^ BDD anode/GDE cathode Current density: 5 mA cm^−2^ [Na_2_SO_4_]: 0.05 M pH: 2.8 [Fe^2+^]: 1 mg L^−1^ Volume: 250 mL	0.606 min^−1^ (1.07 x 10^−2^ s^−1^)	• 43% of mineralization after 120 min of treatment.
AMP [11]	Photo-Electro-Fenton	[AMP]: 50 mg L^−1^ BDD anode/GDE cathode Current density: 5 mA cm^−2^ [Na_2_SO_4_]: 0.05 M pH: 2.8 [Fe^2+^]: 1 mg L^−1^ UVA light: 5.0 W m^−2^ Volume: 250 mL	1.086 min^−1^ (1.81 x 10^−2^ s^−1^)	• 63% of mineralization and Complete AA removal after 120 min of treatment. • Degradation of AMP higher than 90% in a real industrial wastewater from a Slaughterhouse company.
AMP [12]	Non-thermal plasma	[AMP]: 20 mM (6.99 g L^−1^) Plasma was generated using a nanosecond-pulsed power supply with alternating polarity and a floating electrode-dielectric barrier discharge. Samples were treated under atmospheric conditions with no gas flow. The treatment of all samples was at 11.2 kV and 690 fHz	Not reported	• Complete AMP degradation was achieved after 5 min of treatment. • Preliminary product formed is ampicillin sulfoxide; however, many more species are formed as treatment time is increased.
AMP [13]	ZnO photocatalysis	[AMP]: 105 mg L^−1^ [ZnO]: 0.5 g L^−1^ UVA light: 6 W pH: 11.0 Volume: 500 mL	0.015 min^−1^	• 9.7% of mineralization after 180 min of treatment.
AMP [in this work]	Sono-photo-Fenton	[AMP]: 30 μM (10.5 mg L^−1^) [Fe^2+^]: 90 μM (5.0 mg L^−1^) UVC light: 4 W Ultrasound power: 24.4 W Frequency: 375 kHz initial pH: 6.5 Volume: 250 mL	0.1065 min^−1^	• Complete AA removal after 60 min of treatment. • 90.5% of NAF removal in WWTP after 60 min of treatment.
NAF [14]	Electrochemical oxidation	[NAF]: 50 mg L^−1^ BDD anode/GDE cathode Current density: 5 mA cm^−2^ [Na_2_SO_4_]: 0.05 M pH: 2.8 Volume: 250 mL	0.604 min^−1^ (1.00 x 10^−2^ s^−1^)	• ∼50% of AA removal after 90 min of treatment.
NAF [14]	Electro-Fenton	[NAF]: 50 mg L^−1^ BDD anode/GDE cathode Current density: 5 mA cm^−2^ [Na_2_SO_4_]: 0.05 M pH: 2.8 [Fe^2+^]: 1 mg L^−1^ Volume: 250 mL	0.873 min^−1^ (1.46 x 10^−2^ s^−1^)	• ∼60% of AA removal after 90 min of treatment.
NAF [14]	Photo-Electro-Fenton	[NAF]: 50 mg L^−1^ BDD anode/GDE cathode Current density: 5 mA cm^−2^ [Na_2_SO_4_]: 0.05 M pH: 2.8 [Fe^2+^]: 1 mg L^−1^ UVA light: 5.0 W m^−2^ Volume: 250 mL	1.560 min^−1^ (2.60 x 10^−2^ s^−1^)	• Complete AA removal after 90 min of treatment. • A solution of NAF treated during 90 min was coupled to a bio-process leading to 85% of the solution mineralization.
NAF [in this work]	Sono-photo-Fenton	[NAF]: 30 μM (12.4 mg L^−1^) [Fe^2+^]: 90 μM (5.0 mg L^−1^) UVC light: 4 W Ultrasound power: 24.4 W Frequency: 375 kHz initial pH: 6.5 Volume: 250 mL	0.1638 min^−1^	• Complete AA removal after 20 min of treatment. • 96.3% of NAF removal in WWTP after 60 min of treatment.

## 2 Experimental design, materials, and methods

### 2.1 Reagents

Ampicillin trihydrate was provided by Syntopharma. Sodium nafcillin was purchased from Sigma. Sodium chloride, potassium chloride, acetonitrile, urea, nutrient agar, magnesium sulfate heptahydrate and sodium sulfate were purchased from Merck. Dipotassium hydrogen phosphate, sodium bicarbonate, calcium sulfate dihydrate and ferrous sulfate heptahydrate were provided by Panreac. Formic acid from Carlo Erba was used. Peptone and meat extract were purchased from Oxoid. All chemicals were used as received. The solutions of antibiotics were prepared using distilled water.

### 2.2 Reaction systems

A Meinhardt ultrasound reactor was used for sonochemical process operated at 375 kHz and 24.4 W. For the combined system, the ultrasound reactor was complemented by an UVC-lamp (4 W) with main emission at 254 nm (OSRAM G4T5/OF) placed on a quartz sleeve (which was submerged in the aqueous sample). In all cases, the reactor temperature was controlled using a Huber Minichiller.

### 2.3 Analyses

Antibiotics degradation was followed using UHPLC Thermoscientific Dionex UltiMate 3000 instrument equipped with an AcclaimTM 120 RP C18 column (5 μm, 4.6x150 mm) and a diode array detector, through the methods utilized by Vidal et al. [11,14]. Accumulation of hydrogen peroxide was determined by an iodometry-spectrophotometry methodology according to Serna-Galvis et al. [2]. The antimicrobial activity (AA) was determined by measurement of the inhibition zone in the agar diffusion test [15].
